# Domain Adaptation Using Convolutional Autoencoder and Gradient Boosting for Adverse Events Prediction in the Intensive Care Unit

**DOI:** 10.3389/frai.2022.640926

**Published:** 2022-04-11

**Authors:** Yuanda Zhu, Janani Venugopalan, Zhenyu Zhang, Nikhil K. Chanani, Kevin O. Maher, May D. Wang

**Affiliations:** ^1^School of Electrical and Computer Engineering, Georgia Institute of Technology, Atlanta, GA, United States; ^2^Biomedical Engineering Department, Georgia Institute of Technology, Emory University, Atlanta, GA, United States; ^3^Biomedical Engineering Department, Georgia Institute of Technology, Atlanta, GA, United States; ^4^Department of Biomedical Engineering, Peking University, Beijing, China; ^5^Pediatrics Department, Emory University, Atlanta, GA, United States

**Keywords:** intensive care units, clinical decision support, mortality prediction, gradient boosting, convolutional autoencoder, domain adaptation (DA)

## Abstract

More than 5 million patients have admitted annually to intensive care units (ICUs) in the United States. The leading causes of mortality are cardiovascular failures, multi-organ failures, and sepsis. Data-driven techniques have been used in the analysis of patient data to predict adverse events, such as ICU mortality and ICU readmission. These models often make use of temporal or static features from a single ICU database to make predictions on subsequent adverse events. To explore the potential of domain adaptation, we propose a method of data analysis using gradient boosting and convolutional autoencoder (CAE) to predict significant adverse events in the ICU, such as ICU mortality and ICU readmission. We demonstrate our results from a retrospective data analysis using patient records from a publicly available database called Multi-parameter Intelligent Monitoring in Intensive Care-II (MIMIC-II) and a local database from Children's Healthcare of Atlanta (CHOA). We demonstrate that after adopting novel data imputation on patient ICU data, gradient boosting is effective in both the mortality prediction task and the ICU readmission prediction task. In addition, we use gradient boosting to identify top-ranking temporal and non-temporal features in both prediction tasks. We discuss the relationship between these features and the specific prediction task. Lastly, we indicate that CAE might not be effective in feature extraction on one dataset, but domain adaptation with CAE feature extraction across two datasets shows promising results.

## Introduction

Each year, over 5 million patients have admitted to ICUs in the United States (Vranas et al., 2018), with an average mortality between 8 and 10% (Wu et al., 2002). The leading causes of mortality are cardiovascular collapses (Benjamin et al., 2017), multi-organ failures (Marshall et al., 1995), and sepsis (Kissoon et al., 2016). Studies have shown that prolonged hospital stays lead to increased life-threatening outcomes, which are associated with adverse events and other risks in the ICU environment (Celi et al., 2011; Hunziker et al., 2012), such as severe infection (Fagon et al., 1994; Ribas et al., 2011), cardiac arrest (Nemati et al., 2011), extended invasive ventilation (Fagon et al., 1993; de Rooij et al., 2006), mortality within 1 year (Mandelbaum et al., 2011; Celi et al., 2012), ICU/hospital readmissions (Fialho et al., 2012), acute kidney injury (Mandelbaum et al., 2011; Celi et al., 2012), and hypotension (Lee and Mark, 2010; Hug et al., 2011; Lee et al., 2012). Studies also report that these complications will also lead to a significant increase in the costs incurred (Wheeler et al., 2011; Kwon et al., 2012; Hutchinson et al., 2013). Hence, accurate prediction of adverse patient endpoints would allow for improved resource allocation.

Research on the analysis of patient data to predict adverse events, such as ICU mortality and ICU readmission, has mainly used probabilistic models. Logistic regression (Celi et al., 2012; Fuchs et al., 2012; Lee et al., 2012; Venugopalan et al., 2017), Cox regression (Fuchs et al., 2012), and artificial neural networks (Wong and Young, 1998; Lee and Mark, 2010; Celi et al., 2012) are the most common models used in the analysis of healthcare data (Goldstein et al., 2017). However, these models suffer from inherent issues, particularly, their basis on using a snapshot of the data available to make longitudinal predictions. Patient data itself are temporal in nature; hence, a temporal analysis of these data should be performed for a more appropriate health prediction.

The temporal models commonly seen in the literature include models, such as sequence analysis (Wang et al., 2008; Batal et al., 2011; Tao et al., 2012; Casanova et al., 2015; Syed and Das, 2015), association rule mining (Bellazzi et al., 2011; Casanova et al., 2015; Yang and Yang, 2015), temporal Cox regression (Warner et al., 2013; Cai et al., 2015; McCoy et al., 2015), and clustering (Toddenroth et al., 2014; Choi et al., 2017). Sequence analysis and association rule mining-based studies require extensive user input for identifying specific features whose patterns of correlation can be studied with respect to the target variable. In addition, they are not amenable for discerning relationships and patterns contributing to adverse events, from a large number of features. Regression- (Singh et al., 2015) and clustering-based (Doshi-Velez et al., 2014) studies use the information within a specific time interval for analysis. These studies do not account for the differing length of available data for different patients. Cox regression also does not account for the dependency between the consecutive time points. Graphical methods by Liu et al. use Gaussian Processes (GPs) for time-series analysis (Liu et al., 2013). Their assumption is that the data are piecewise linear and use only the GP coefficients for classification. Such models make the assumptions that ICU data can be approximated using piecewise GPs. Stiglic et al. (2013) used past recordings for a single patient to make predictions about a future time instant using the least absolute shrinkage and selection operator (LASSO) regression. The parameters of these models are trained for each individual patient and do not make use of the information, which can be learned from large databases consisting of multiple patients. Such models not only require the user to train the model for each patient but also tend to over-fit the data. In addition, these models do not tell the clinicians if the patients are improving over time. Yu et al. (2011) generates individual survival curves by using a series of logistic regression models to calculate the hazard at each time instance. This approach is not only very computationally intensive but also does not account for the variation in the duration of ICU data.

Transfer learning has been widely used in different clinical decision support systems, especially on medical image processing tasks (Han et al., 2018; Lee et al., 2018; Cheplygina et al., 2019; Choudhary et al., 2020). The goal of transfer learning is to use an external dataset to improve classification or segmentation results on the local dataset. There are two major concepts related to transfer learning: domain and task. Medical images are considered from different domains if they are generated by different scanners or follow different scanning protocols. Therefore, datasets collected from multiple sites or providers are considered from different domains. Task refers to the specific machine-learning task, such as disease classification (Chen et al., 2017; Hussein et al., 2017; Li et al., 2018), lesion detection (Hwang and Kim, 2016; Elmahdy et al., 2017; Murthy et al., 2017), and tumor segmentation (Kandemir, 2015; Huynh et al., 2016).

One category of transfer learning is “same domain, different tasks.” Studies in this category would use the same dataset for multiple tasks. A common strategy is feature transfer for multi-task learning that proposed models aim to learn common features across different tasks. Joint learning on task-independent features can effectively mitigate the overfitting problem by regularizing the classifiers and/or increasing sample size. Another category of transfer learning is “different domains, same task.” Imaging data collected from different domains assumably have different sample data distribution; hence, domain adaptation is needed. The objective of domain adaptation is to transfer the knowledge across different domains by learning domain-invariant features transformation. Image-to-image domain transformation (Isola et al., 2018) achieves pixel-level mapping between source and target images using generative models, such as generative adversarial networks (GAN) (Goodfellow et al., 2014). Another approach is latent feature space transformation that learns the domain-invariant features representation by transforming source and target domain images into the shared latent space. Latent feature space transformation has three subcategories of methods: reconstruction-based (autoencoder) (Bousmalis et al., 2016; Ghifary et al., 2016), divergence minimization (divergence metric) (Damodaran et al., 2018; Kang et al., 2019; Rozantsev et al., 2019), and adversarial learning (discriminator) (Lafarge et al., 2017).

Despite its success in image processing (Han et al., 2018; Lee et al., 2018; Choudhary et al., 2020), transfer learning has a limited impact on ICU data for predicting mortality risk and ICU readmission. Gupta et al. (2020) proposed multi-task transfer learning using recurrent neural network (RNN) on Multi-parameter Intelligent Monitoring in Intensive Care (MIMIC)-III data to identify phenotypes and predict in-hospital mortality. The proposed RNN is pretrained on predicting phenotypes, before transferring the learned knowledge on the mortality prediction task. Although the author demonstrated the effectiveness of multi-task transfer learning, there are two shortcomings of the work: (1) the proposed RNN model is close to a vanilla model. The complicated RNN models, such as gated recurrent units (GRUs) or long short-term memory (LSTM), could be used to improve the pipeline. (2) There is no preprocessing on the MIMIC-III data, as the author directly worked on the benchmark data. Another way to do transfer learning is to train the model on two datasets to improve prediction performance. Desautels et al. (2017) concatenated MIMIC-III data (external dataset) with UK ICU data (internal dataset) for joint training before evaluating the model on the internal testing set. Multi-source transfer learning is novel on ICU adverse outcome prediction, but the proposed model suffers from very low specificity (0.5917) and poor f1-score (0.1321).

In this study, we propose a retrospective study of adult populations to discover factors indicative of adverse events, such as ICU mortality and 30-day ICU readmissions, using a gradient boosting algorithm for classification and convolutional autoencoder (CAE) for domain adaptation. We apply CAE as domain adaptation for learning domain-invariant latent feature representation from two ICU datasets. We aim to improve model classification performance on our internal dataset after learning from the external dataset.

We summarize our major contribution in several folds:

- We demonstrate that gradient boosting is effective in both the mortality prediction task and the ICU readmission prediction task.
- We use gradient boosting to identify top-ranking temporal and non-temporal features in both the mortality prediction task and the ICU readmission prediction task. We discuss the relatedness of these features with their corresponding prediction task.
- We indicate that CAE might not be effective in feature extraction on one dataset, but domain adaptation with CAE feature extraction across two datasets shows promising results.

We structure the remainder of this article as follows. First, a short description of our data source is followed by a detailed description of the preprocessing and data mining approaches in Section Materials and Methods. Experiments are described in Section Experiments. Results and discussion are presented in Section Results and Discussion. Finally, the conclusion and future directions are summarized in Section Conclusion.

## Materials and Methods

In this study, we performed the classification of ICU patients into high risk and low risk for adverse events using random forest, gradient boosting, and CAE. We demonstrated our results using a retrospective data analysis of adult ICU data from the MIMIC-II database and local ICU data from Children's Healthcare of Atlanta (CHOA). We used different machine-learning models to determine patient's factors, which contribute to adverse consequences, such as ICU mortality and 30-day ICU readmission. These endpoints are particularly interesting since they provide the basis for the long-term prediction of adverse events.

### Data and Preprocessing

#### MIMIC Data

Multi-parameter Intelligent Monitoring in Intensive Care-II is a public ICU data repository containing over 40,000 ICU stay (32,331 adults and, 8,085 neonatal) records (Saeed et al., 2011). We performed retrospective data analysis using adult patient data (>16 years). Each ICU stay record consists of the patient's demographic information, diagnosis, chart events, medication intake events, microbiology events, etc. (example features in Table 1). Each patient record consists of features which are either static (does not change over the entire duration of the patient's ICU stay) or temporal (changing in time). From the total of over 13,000 features, we ranked the features by the frequency of measurement. Using the top 2,000 most frequent features, we picked 87 features on the basis of clinician judgment (we used only 84 features for ICU readmission to avoid information leakage). The included features that covered clinical measurements, lab results administrative data, comorbidities, and other diagnostic procedures.

**Table 1:** Summary of Multi-parameter Intelligent Monitoring in Intensive Care (MIMIC) and Children's Healthcare of Atlanta (CHOA) data.

**Data and features**	**MIMIC II**	**CHOA**	**Data type**
Sample size	40,416 patient records	5,739 patient records	
Demographics	Gender, age, height, weight, ethnicity, comorbidity	DOB, gender, age, height, weight, ethnicity, religion, date of death, co morbidity with other diseases	Non-temporal
Lab data	Urea, albumin, bilirubin, creatinine, sodium, potassium, calcium	Urea, albumin, bilirubin, creatinine, sodium, potassium, calcium	Temporal
Chart data	Heart rate, blood pressure, NBP, CVP, SaO_2_, arterial PH, arterial PaCO_2_, arterial PaO_2_		Temporal
Microbiology		Types of microbes, amount of microbes, dilution	Non-temporal
Medication data		Medication and IV administered, dosage, duration time, concentrations and rate of administration, composition of IV imposed	Non-temporal

For this analysis, the data from each feature were binned into binning intervals of 6 h. After binning the data into intervals of 6 h, data had 87 ± 21% of missing data. The missing data were divided into multiple types of missing data known in the statistical literature as “missing completely at random (MCAR),” “missing at random (MAR),” and “missing not at random (MNAR).” Then each type was imputed differently using the techniques described in a previous paper on data imputations (Venugopalan et al., 2019). MNAR data were imputed using Student's t-copulas and MAR data were imputed using expectation maximization (EM) after clustering.

We then proceeded to perform feature selection and classification on the patient data sequences for identifying patients at risk for adverse events, such as mortality in the ICU and 30-day ICU readmission. In this dataset, there were 2,334 patient records with mortality during the ICU stay and 29,997 patient records of successful discharge from the ICU. Similarly, 7,787 patients' records had ICU readmission within 30 days and 24,544 patients did not relapse into the ICU within 30 days.

#### CHOA Data

The other dataset is from CHOA containing 5,739 patient records spanning an 11-month period. The visits spanned pediatric ICU, neonatal ICU, and cardiac ICU. As shown in Table 1, each ICU stay record consists of the patient's demographic information (e.g., gender and age of admission), diagnosis [e.g., International Classification of Diseases (ICD-9) codes], birth-related events (e.g., birth weight, head circumference, gestation weeks), microbiology events (e.g., microbes in blood or serum), chart events (e.g., heart rate), medication intake events, and clinical records (e.g., pulse oximetry) collected from bedside monitors, averaged over each minute.

The data columns are binary, categorical, and quantitative from which we extracted features (9,071 non-temporal and 2,500 time-series features). To be more specific, we used categorical data, such as the disease codes and procedure codes, into the number of times each disease or condition was presented or the procedure performed. This gives us 9,071 non-temporal features that consist of demographics, microbiology, diagnosis codes, and medication data. In this dataset, since the temporal information for microbiology, medication, and pathology was not available, we treated them as non-temporal data and performed aggregates over the duration of the stay. The temporal data we used were from the various lab tests performed. Lab test data had a median sampling interval of 2.05 h, from which we extracted 2,500 features. After removing features with >80% missing data, we were left with 1,882 lab features, which we binned into 2 h binning intervals. In addition, this dataset had an issue where the tests or values were not recorded for very long-time intervals (≈several days) in the middle. This could be due to the fact that the patients were no longer in the ICU. We treated these type of data as multiple time-series for each patient visit and did not use the missing period for binning. This gave us a total of 8,489 series. Since non-temporal data have <1% data missing, no features were removed.

The features extracted in the previous step were either quantitative real numbers or binary. The range of quantitative features had an order of magnitude variation (e.g., respiration rate varied from 10 to 30 breaths per min, blood pressure varied from 90 to 150 mmHg, and blood calcium varied between 8 and 11 mg/dl). To address this issue, we normalized all the features between the ranges 1 and 2. We also converted the binary values into 1 or 2.

### Classifiers

Four classifiers are introduced in this section. Gradient boosting and random forest are ensemble models of decision trees. We will also briefly describe the linear regression classifier and support vector machine.

#### Random Forest

A random forest classifier is an ensemble of decision trees that take the advantage of a large number of uncorrelated trees. Each decision tree in the random forest makes a class prediction and the random forest model takes the votes of the majority. The core theory behind random forest is that a large crowd of decision trees would outperform individual trees. To ensure model generalizability, random forest enables feature randomness that when splitting a node, the model considers all possible features and selects the one leading to the largest separation between left node observations and right node ones. In this work, we used the online package Sklearn^1^ to apply the random forest classifier. We used Gini impurity as the criterion to measure the quality of split. To mitigate the overfitting issue, the maximum tree depth was used; it was also the hyperparameter that we tuned in an experiment using grid search.

#### Gradient Boosting

Boosting is to convert weaker learners into stronger learners. Typically, a weaker learner is a decision tree that classifies the data with a poor performance. Each new tree in boosting is a fit to a modified copy of the original dataset. Boosting can be explained as a numerical optimization problem that the objective is to minimize the loss function defined in the model by using a gradient descent procedure when adding new weak learners.

Gradient boosting (Friedman, 2001) is a stage-wised additive model. It trains many models in a sequential and gradual way. When a new weak learner is added, existing weak learners remain unchanged in the model. Gradient boosting allows the optimization of arbitrary loss functions that are differentiable. In this work, we used the online package Sklearn to apply gradient boosting classifier on ICU data. We used deviance as the loss function, and Friedman mean squared error (Friedman, 1997) to measure the quality of tree split. To mitigate overfitting issue, the maximum tree depth is used; it was also the hyperparameter that we tuned in an experiment using grid search.

#### Linear Regression

A Linear regression classifier of Ordinary Least Squares fits a linear model that aims to minimize the residual sum of squares between the prediction (linear approximation) and the ground truth observations. The mathematical formula is: $\underset{w}{\text{min}}{\left||Xw-y\;|\right|}_{2}^{2}$.

Where X is the input data matrix, w is the weight matrix, and y is the predicted vector. A major limitation of the linear regression classifier is that it is sensitive to class imbalance. We have used linear regression as a baseline model in this work.

#### Support Vector Machine

Support vector machine is a kernelized method for classification and outlier detection. It is effective for high-dimensional feature data. In this project, we have used the Radial Basis Function (RBF) kernel for our SVM classifier.

### CAE and Domain Adaptation

**Figure 1:**
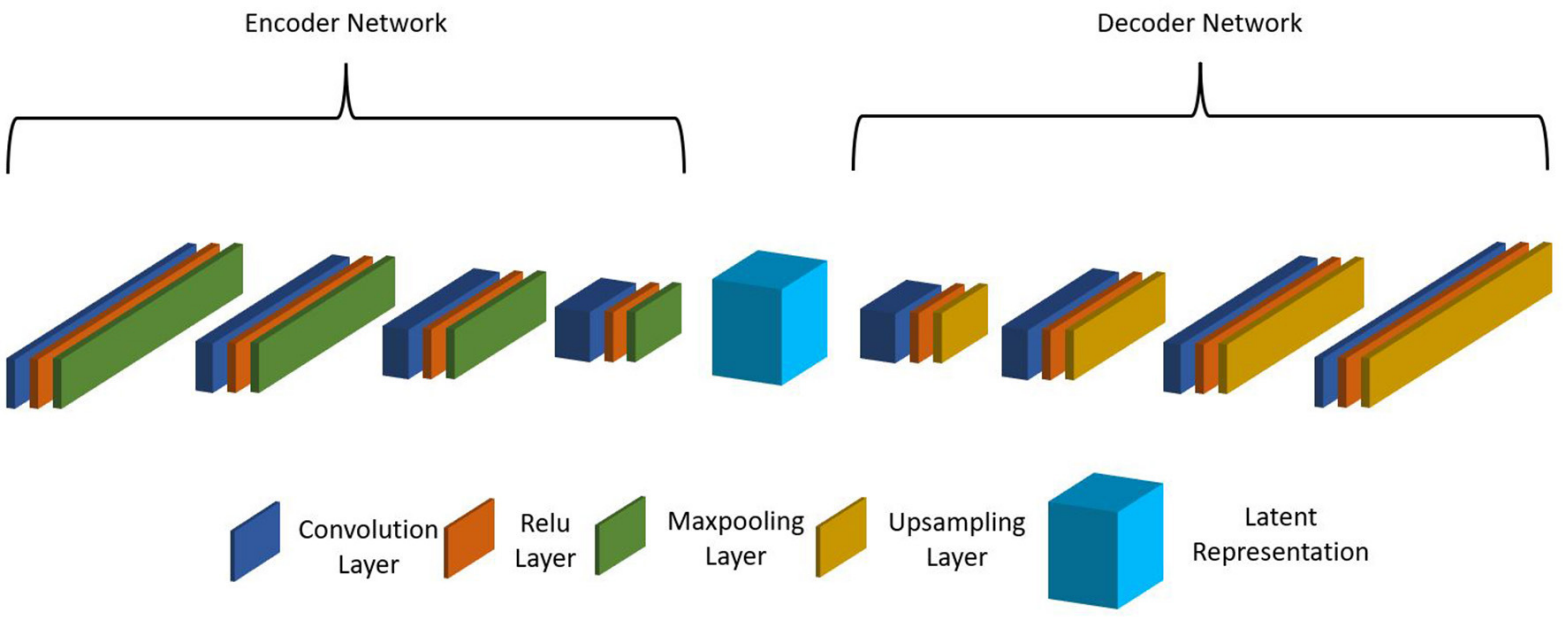
Structure of proposed convolutional autoencoder.

As the MIMIC dataset has many more patient records than the internal CHOA dataset, we would like to transfer the knowledge learned from the external dataset and apply to the internal dataset to improve model prediction performance. Specifically, we would like to apply domain adaptation to transform data from both domains into a shared feature space and learn domain-invariant feature representation. When inspecting the datasets, we found that the feature names in MIMIC and CHOA datasets cannot be matched since the two datasets are not following the same rules to name their features. This is a major challenge, as we cannot directly learn the domain-independent features across the two datasets. Thus, we proposed to use non-negative matrix factorization (NMF) and CAE for latent feature space transformation. NMF reduces feature dimension so that the CAE model can fit on data from both domains. CAEs are effective in learning latent feature vectors on pathological whole slide images (Zhu et al., 2019); yet for this project, we used CAE with 1D convolutional layers. As shown in Figure 1, the stacked CAE consists of an encoder that encodes the original input feature vector into compressed feature representation and a symmetric decoder that projects learned feature representation onto the original feature space. Each block of the encoder includes a 1D convolutional layer, a ReLu activation layer, and a max pooling layer. Similarly, each block of the decoder includes a 1D convolutional layer, a ReLu activation layer, and an upsampling layer. The reconstructed output vector is compared with the original input vector, and the loss is computed and backpropagated during optimization.

## Experiments

**Figure 2:**
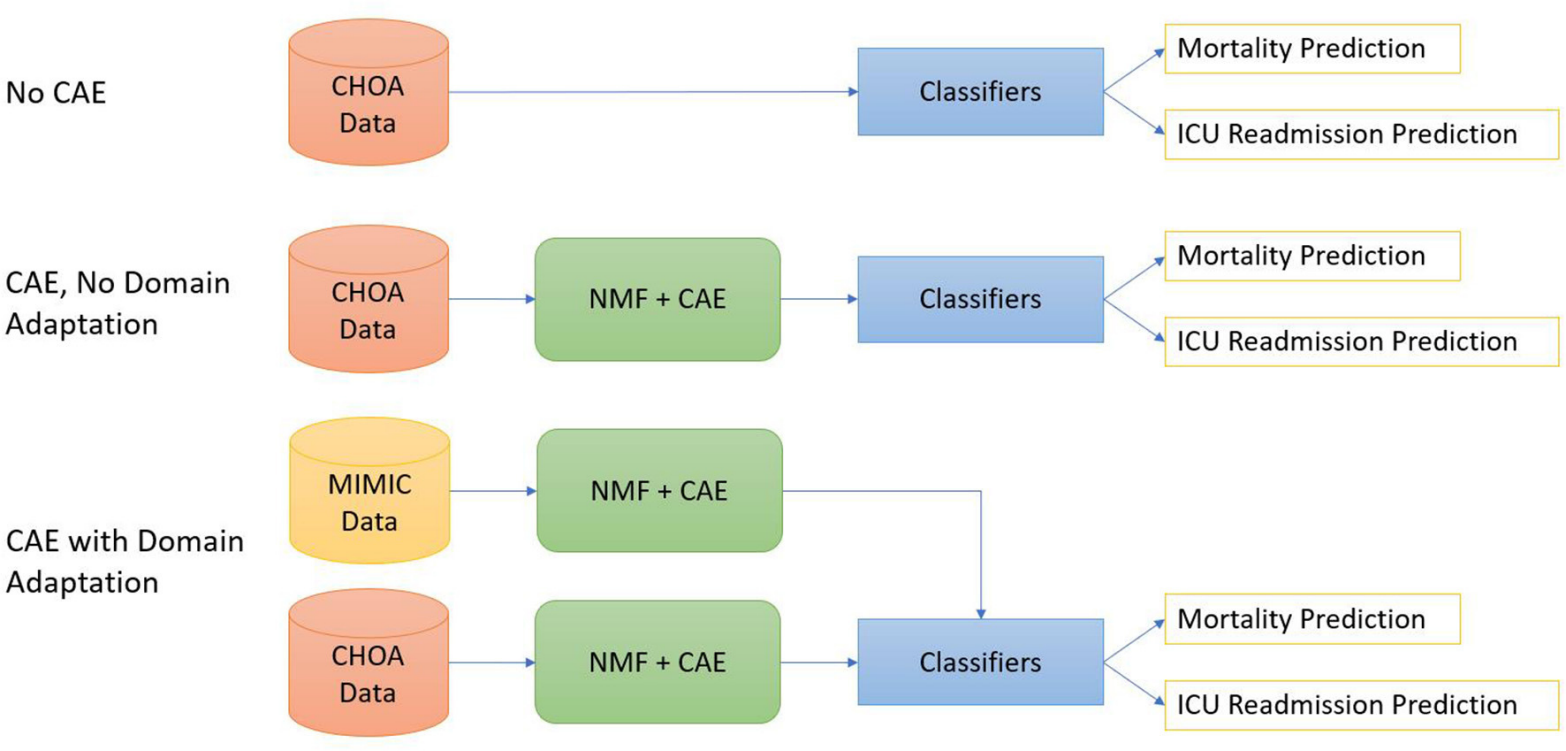
Diagrams for three experiments settings. The top one is to apply classifiers directly on the Children's Healthcare of Atlanta (CHOA) data. The middle one is to apply non-negative matrix factorization (NMF) and convolutional autoencoder (CAE) on CHOA without domain adaptation. The bottom one is domain adaptation using CAE.

As shown in Figure 2, we design three different experimental settings for both mortality prediction and ICU readmission prediction.

1. *Shallow classifiers only*. We directly applied shallow classifiers, such as gradient boosting, random forest, linear regression, and SVM models, on temporal and non-temporal features of CHOA data.
2. Convolutional autoencoder *and shallow classifiers*. We first applied NMF on CHOA data for feature dimensionality reduction and then used a CAE model to learn latent feature representation. Afterward, we applied shallow classifiers on the concatenation of temporal and non-temporal features of the CHOA data.
3. Convolutional autoencoder*, domain adaptation, and shallow classifiers*. We first separately applies NMF on MIMIC and CHOA data for feature dimensionality reduction, then used two separate CAE models to learn latent feature representation from these two datasets. We then pretrain shallow classifiers on the learned latent feature vectors of MIMIC data and fine-tuned the classifiers on CHOA features before evaluating the testing set of CHOA data.

Hyperparameter tuning was conducted using grid search and cross validation. The best classification model was automatically selected based on the highest average AUC-ROC score across 5-fold cross-validation. The maximum tree depth was the hyperparameter tuned for the random forest model and gradient boosting model. We also tried a different number of components in NMF. As for CAE, we empirically varied the number of blocks between 3 and 5. The number of filters in each convolutional layer increases by a factor of 2 from outside to the inside of the encoder, the kernel size of the convolutional layer is 2, and the stride is 1. The CAE was implemented in Keras/TensorFlow.

We performed statistical analysis using two-way analysis of variance (ANOVA) to compare classification results. The two independent variables are experiment settings and classifiers. The null hypothesis is that the mean classification results in different groups are the same. Thus, the alternative hypothesis is that one group mean is different from other groups. In addition to variable-level statistical analysis, we also tried to identify which values of these two variables are significant. We performed a pairwise comparison using the Tukey's honestly significant difference (HSD) test (Keselman and Rogan, 1978). We would reject the null hypothesis if the *p* is <0.05.

## Results and Discussion

### Temporal and Non-Temporal Features

We first evaluated the classification performance using different feature sets. As shown in Table 2 and Table 3, the highest area under the curve (AUC)-receiver operating characteristic (ROC) score is achieved using a gradient boosting classifier on the concatenation of both temporal and non-temporal features. On the mortality prediction task, both feature sets have better performance than temporal only feature sets, which are better than non-temporal only feature sets using gradient boosting or random forest classifier. On ICU readmission prediction, the performance between using both feature sets and non-temporal features only is close when using gradient boosting and random forest models; both sets have better performance than temporal feature only. Linear regression and SVM classifiers have lower AUC-ROC scores when using both feature sets than the decision-tree-based models. We argue that non-temperature feature sets and temporal feature sets contain similar information on adverse events prediction, while the concatenation of both feature sets would achieve the best performance.

**Table 2:** Average and standard deviation (SD) of AUC-ROC score for mortality prediction using shallow classifiers with temporal features only, non-temporal features only, and both types of features.

**Classifier**	**Both features**	**Temporal only**	**Non-temporal only**
Gradient boosting	0.95718 (0.01546)	0.91020 (0.03810)	0.90132 (0.01824)
Random forest	0.94583 (0.01982)	0.92798 (0.03218)	0.87685 (0.02455)
Linear regression	0.71654 (0.08129)	0.80372 (0.05604)	0.74497 (0.06087)
SVM	0.62956 (0.02939)	0.62049 (0.03315)	0.78573 (0.04997)

*The highest average score is highlighted in bold and underline*.

**Table 3:** Average and standard deviation (SD) of AUC-ROC score for ICU readmission prediction results using shallow classifiers with temporal features only, non-temporal features only, and both types of features.

**Classifier**	**Both features**	**Temporal only**	**Non-temporal only**
Gradient boosting	0.76651 (0.03185)	0.60214 (0.03058)	0.75414 (0.03747)
Random forest	0.74103 (0.03934)	0.60882 (0.01912)	0.74421 (0.04731)
Linear regression	0.55374 (0.01909)	0.60293 (0.02486)	0.70807 (0.03570)
SVM	0.54261 (0.03597)	0.46318 (0.01806)	0.66418 (0.04506)

*The highest average score is highlighted in bold and underline*.

### Mortality Prediction

**Figure 3:**
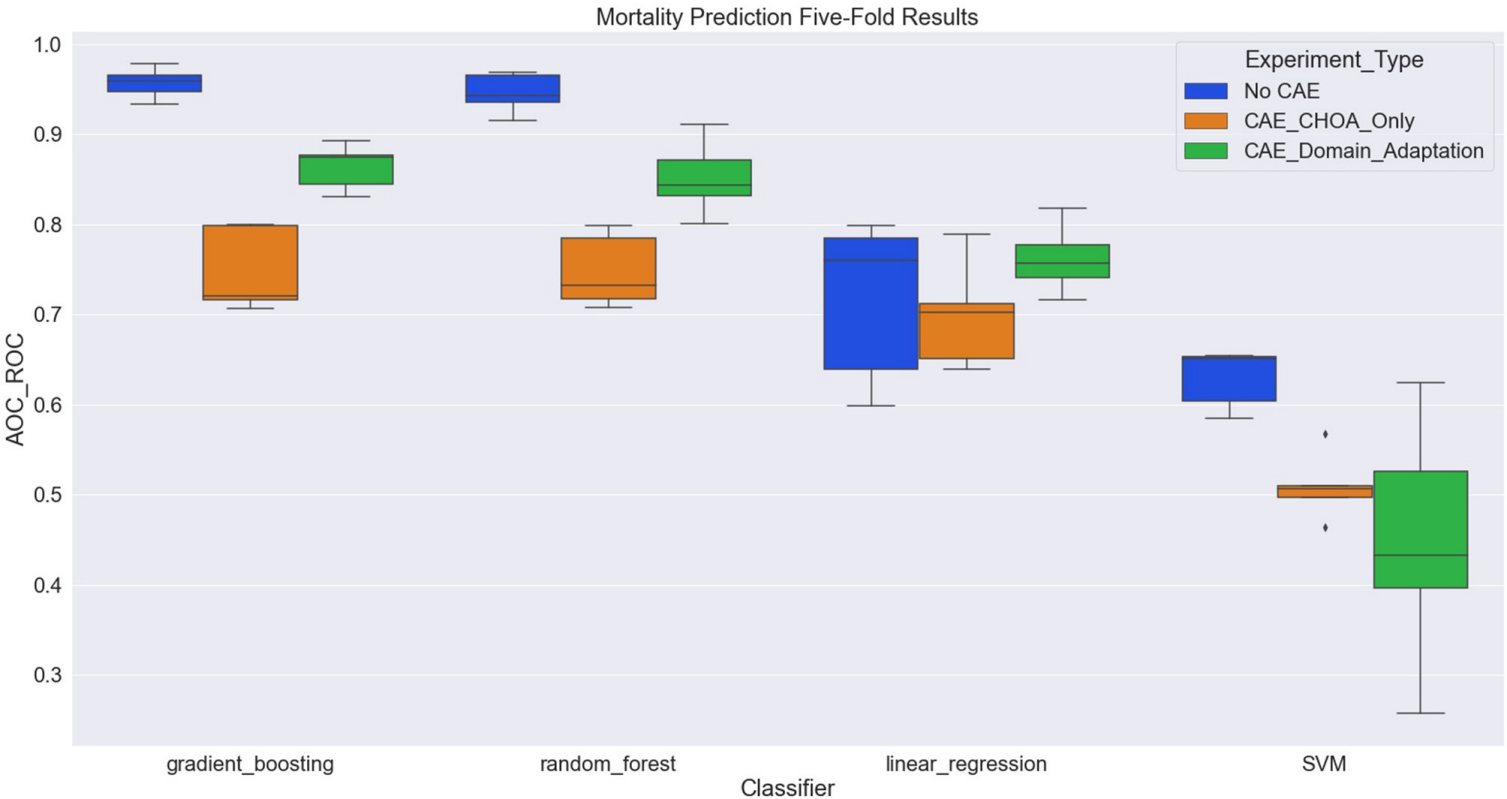
Boxplot of mortality prediction results using four different classifiers with three experiment settings.

Table 4 shows the average and standard deviation (SD) of the mortality prediction results across five-fold on CHOA data under three different experimental settings. We also visualize the results using a box plot in Figure 3. Specifically, the experiment setting of no domain adaptation (directly applying shallow classifiers on CHOA data) achieves better results than the other two experiment settings (pairwise comparison, *p* < 0.05). Domain adaptation using CAE achieves better results than CAE without domain adaptation (*p* < 0.05). This indicates that even though the existing latent feature extraction method using CAE is not effective, domain adaptation between two datasets can significantly improve the performance. Meanwhile, gradient boosting and random forest algorithms are not significantly different in performance (*p* > 0.05), but either of them is better than SVM and linear regression classifier (pairwise comparison, *p* < 0.05).

**Table 4:** Mortality prediction results on and Children's Healthcare of Atlanta (CHOA) data (both temporal and non-temporal features) under three different experimental settings.

**Classifier**	**No domain adaptation**	**CAE on CHOA only**	**CAE and domain adaptation**
Gradient boosting	0.95718 (0.01546)	0.74864 (0.04162)	0.86375 (0.02267)
Random forest	0.94583 (0.01982)	0.74855 (0.03668)	0.86375 (0.02267)
Linear regression	0.71654 (0.08129)	0.69893 (0.05303)	0.76208 (0.03429)
SVM	0.62956 (0.02939)	0.50896 (0.03349)	0.44725 (0.12380)

*The highest average score is highlighted in bold and underline*.

We also identify the top features for MIMIC and CHOA mortality prediction that include both temporal information and non-temporal information (in Table 5). For the MIMIC dataset, maximum and minimum values of Simplified Acute Physiology Score (SAPS) (Agha et al., 2002) and Sequential Organ Failure Assessment (SOFA) (Arts et al., 2005) score are top-ranking features. SAPS II and SOFA are clinical scores that were designed to assess the severity of illness for ICU patients and to predict their risk of mortality, using lab tests and clinical data. Features, such as hospital length of stay and ICU length of stay, might be controversial for mortality prediction, as the longer length of stay may indicate more severe illness and they fail to predict “ahead of mortality event.” For CHOA dataset, the top-ranking features are lab measurements, clinical events and drug information and ventilator days. These top-ranking features are domain-specific; they are very different from the features in the MIMIC dataset. Further validation and interpretation of these top-ranking features are needed to identify potential biomarkers for clinical practice.

**Table 5:** Top 10 features in mortality prediction using gradient boosting on Multi-parameter Intelligent Monitoring in Intensive Care (MIMIC) and Children's Healthcare of Atlanta (CHOA) data.

**Feature ranking**	**MIMIC**	**CHOA**
1	sofa_max	component_name_art base deficit
2	sofa_min	dx_code_v49.86
3	hospital_los	component_name_nrbc
4	icustay_los	component_name_patient fi02
5	sapsi_max	component_name_plasma free hgb
6	sapsi_min	ventilator_days
7	subject_icustay_total_num	dx_rank
8	cost_weight	component_name_ast (sgot)
9	sofa_first	dx_present_on_admit_yn
10	peptic_ulcer	value_in_range_yn

### ICU Readmission Prediction

**Figure 4:**
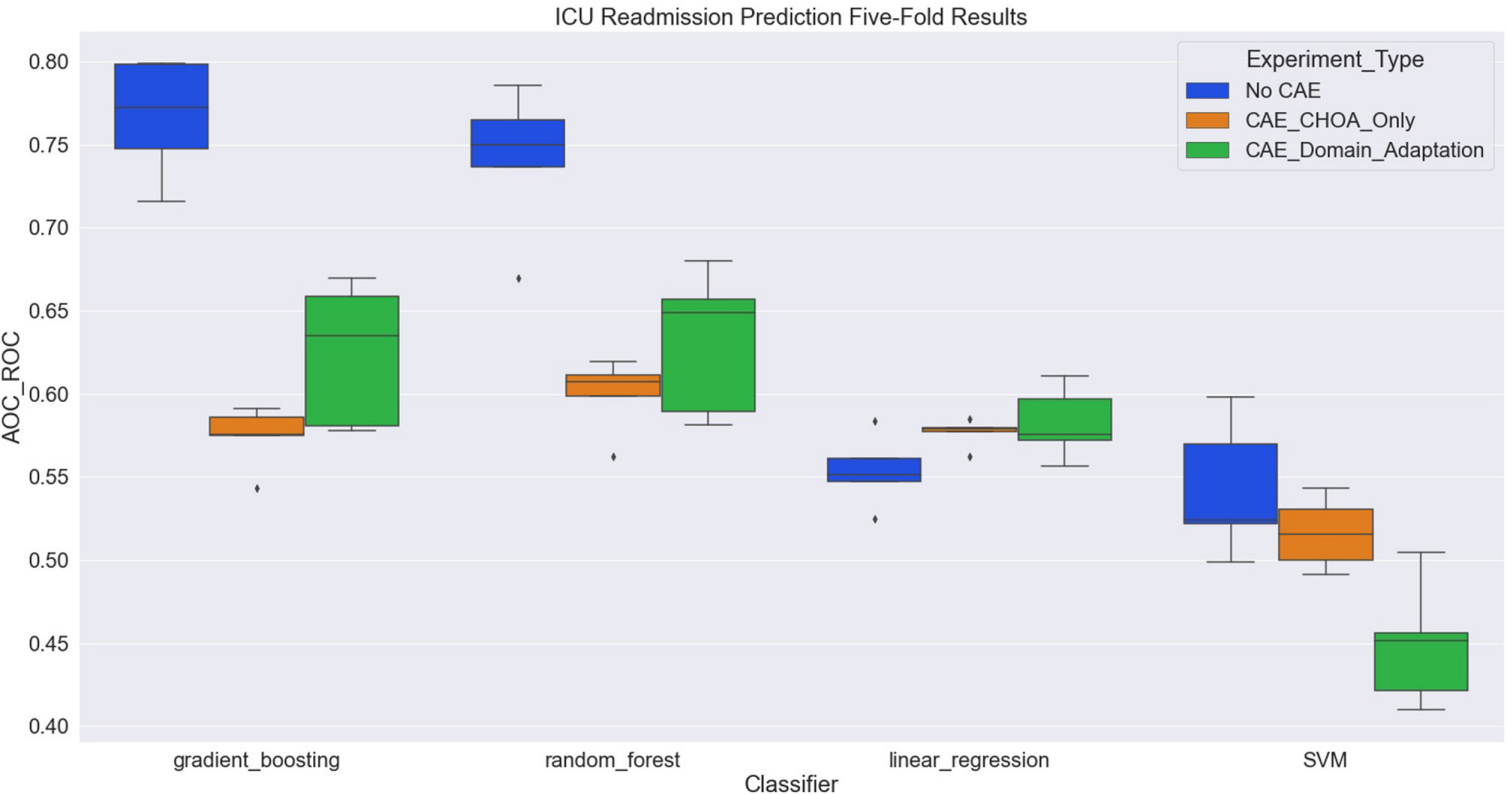
Boxplot of intensive care unit (ICU) readmission prediction results using four different classifiers with three experiment settings.

Table 6 shows the average and SD of the ICU readmission prediction results across 5-fold on CHOA data under three different experimental settings. We also visualize the results using a box plot in Figure 4. Similar to the results in the mortality prediction task, the experiment setting of no domain adaptation (directly applying shallow classifiers on CHOA data) achieves better results than the other two experiment settings (pairwise comparison, *p* < 0.05). However, domain adaptation using CAE fails to achieve better results than CAE without domain adaptation (*p* > 0.05). If we only focus on the best results models (gradient boosting and random forest), domain adaptation has better performance than CAE without domain adaptation (*p* < 0.05). This indicates that even though the existing latent feature extraction method using CAE is not effective, domain adaptation between two datasets can significantly improve the performance. Similar to the results in the mortality prediction task, gradient boosting and random forest algorithms are not significantly different in performance (*p* > 0.05), but either of them is better than SVM and linear regression classifier (pairwise comparison, *p* < 0.05).

**Table 6:** ICU readmission prediction results on Children's Healthcare of Atlanta (CHOA) data (both temporal and non-temporal features) under three different experimental settings.

**Classifier**	**No domain adaptation**	**CAE on CHOA only**	**CAE and domain adaptation**
Gradient boosting	0.76651 (0.03185)	0.57404 (0.01665)	0.62448 (0.03845)
Random forest	0.74103 (0.03934)	0.59993 (0.01988)	0.63117 (0.03904)
Linear regression	0.55374 (0.01909)	0.57664 (0.00749)	0.58234 (0.01914)
SVM	0.54261 (0.03597)	0.51617 (0.01912)	0.44870 (0.03285)

*The highest average score is highlighted in bold and underline*.

Different from mortality prediction task, the top-ranking features for ICU readmission prediction on MIMIC data do not have SAPS II or SOFA scores. As shown in Table 7, these top-ranking features include lab measurements, chart events, and demographic information. We argue that gradient boosting is effective in identifying top-ranking features for both mortality prediction and ICU readmission prediction, as statistical features of SAPS II and SOFA scores are the majority of top-ranking features in the mortality prediction task, but not in the ICU readmission prediction task. For CHOA data, the top-ranking features are different from those in the mortality prediction task, focusing on drug-related features, but still include lab measurements and clinical events. Some of these top-ranking features are pediatric-specific; they are very different from the features in the MIMIC dataset. Similar to the mortality prediction task, further validation and interpretation on these top-ranking features are needed to identify potential biomarkers for clinical practice.

**Table 7:** Top 10 features in ICU readmission prediction using gradient boosting on Multi-parameter Intelligent Monitoring in Intensive Care (MIMIC) and Children's Healthcare of Atlanta (CHOA) data.

**Feature ranking**	**MIMIC**	**CHOA**
1	Gender	dx_code_v44.1
2	hospital_los	dx_rank
3	tidal volume (obser)	apgar_score_5_minutes
4	temperature f	gestation_age_weeks
5	icustay_los	dx_type_coded final
6	spo2	nicu_yn
7	carbon dioxide	birth_weight
8	renal_failure	discharge_destination
9	congestive_heart_failure	dx_present_on_admit_exempt_yn
10	fingerstick glucose	birth_length

## Conclusion

In this work, we extracted temporal and non-temporal features from one public ICU dataset (MIMIC) and a local ICU dataset (CHOA) to build predictive models on mortality risk and ICU readmission. We designed three different experimental settings, implemented CAE to learn latent feature representation, and applied multiple classifiers, such as gradient boosting and random forest for classification. We demonstrated the effectiveness of gradient boosting in both mortality prediction and ICU readmission prediction tasks. In addition, we showed that domain adaptation using CAE across two datasets can significantly improve results against using CAE and classifiers without domain adaptation. We aim to learn domain-invariant latent feature representation and improve prediction performance on the clinical adverse event when the local data set has a very limited sample size.

There are some limitations of this work. First, the temporal features in the CHOA dataset are binned into intervals of 6 h each, which could result in loss of granularity of data and introduction of new missing data points. Second, domain adaptation is designed on the latent feature representation level, not on the deep neural network (CAE) level. This is largely due to the different feature names and feature quantities, as the same deep neural network model with the same hyperparameters cannot be used on both datasets. Third, there is a fundamental bias between the two datasets. For the MIMIC dataset, we extracted ICU data for patients over 16 years old; for the CHOA dataset, we expect the majority of the patients is children. Consequently, the knowledge learned from the adult patient group may not help the model predictions on the children patient group.

As for future work, we would like to acquire time-stamped temporal information for the CHOA ICU dataset. We will implement deep learning models, such as RNNs, to capture the temporal information of lab tests and microbiology events (instead of aggregating them into non-temporal data). We believe that a combination of static and temporal models could give additional insight into the disease process and improve prediction performance. In addition, we will apply fairness-learning techniques to mitigate biased prediction on age- and birth-related factors. In this way, we can improve the fairness of the domain adaptation model and improve the prediction results. Lastly, we would like to overcome the different feature names of the two ICU datasets so that we can apply the same deep neural network to them to transfer the knowledge between the deep neural network.

## Data Availability Statement

The original contributions presented in the study are included in the article/supplementary material, further inquiries can be directed to the corresponding author/s.

## Author Contributions

YZ designed the experiments, implemented the gradient boosting classifier and convolutional autoencoder (CAE), and drafted the article. JV initiated the project, cleaned and preprocessed the data, and helped draft the article. ZZ helped preprocess the data and literature review. NC and KM annotated the CHOA data and provided clinical guidance on top features. MW guided and oversaw the project and reviewed the manuscript. All authors contributed to the article and approved the submitted version.

## Funding

This project was supported in part by the Children's Healthcare of Atlanta (CHOA), the NIH National Center for Advancing Translational Sciences UL1TR000454, the National Science Foundation Award NSF1651360, Microsoft Research, Georgia Institute of Technology PACE, and Hewlett Packard.

## Conflict of Interest

The authors declare that the research was conducted in the absence of any commercial or financial relationships that could be construed as a potential conflict of interest.

## Publisher's Note

All claims expressed in this article are solely those of the authors and do not necessarily represent those of their affiliated organizations, or those of the publisher, the editors and the reviewers. Any product that may be evaluated in this article, or claim that may be made by its manufacturer, is not guaranteed or endorsed by the publisher.

## Footnotes

^1^ https://scikit-learn.org/stable/modules/generated/sklearn.ensemble.GradientBoostingClassifier.html
